# The Role of Hippocampal Replay in Memory and Planning

**DOI:** 10.1016/j.cub.2017.10.073

**Published:** 2018-01-08

**Authors:** H. Freyja Ólafsdóttir, Daniel Bush, Caswell Barry

**Affiliations:** 1 Research Department of Cell and Developmental Biology, UCL, Gower Street, London, WC1E 6BT, UK; 2 UCL Institute of Cognitive Neuroscience and UCL Institute of Neurology, 17 Queen Square, London, WC1N 3AZ, UK

## Abstract

The mammalian hippocampus is important for normal memory function, particularly memory for places and events. Place cells, neurons within the hippocampus that have spatial receptive fields, represent information about an animal’s position. During periods of rest, but also during active task engagement, place cells spontaneously recapitulate past trajectories. Such ‘replay’ has been proposed as a mechanism necessary for a range of neurobiological functions, including systems memory consolidation, recall and spatial working memory, navigational planning, and reinforcement learning. Focusing mainly, but not exclusively, on work conducted in rodents, we describe the methodologies used to analyse replay and review evidence for its putative roles. We identify outstanding questions as well as apparent inconsistencies in existing data, making suggestions as to how these might be resolved. In particular, we find support for the involvement of replay in disparate processes, including the maintenance of hippocampal memories and decision making. We propose that the function of replay changes dynamically according to task demands placed on an organism and its current level of arousal.

## Teaser

Olafsdottir *et al.* explore the involvement of replay in consolidating hippocampal memories and guiding decision making, arguing that distinct forms of replay — which can switch dynamically — support these two functions.

## Main Text

### Introduction

The hippocampus is placed firmly at the centre of a network supporting memory function 1, 2, 3, 4, 5. In humans, hippocampal damage is associated with wide-ranging impairments in autobiographical memory 1, 6 as well as profound deficits in spatial memory, manifesting as a loss in the ability to navigate flexibly through the world [7], although some spatial knowledge can be retained [3]. In rodents, lesions made to the hippocampus and associated structures have generated complementary results, including deficits in spatial working memory 8, 9, impairments in navigation to hidden spatial goals 10, 11, and a more general failure to recognise familiar environments 12, 13.

However, the hippocampus is far from being a simple static repository of past experiences. In patients, memory for distant events can be preserved even when that for more recent events is disrupted by hippocampal damage 1, 14, 15. This temporally graded retrograde amnesia has been taken as evidence that, with time, some memories become less dependent on the hippocampus and increasingly dependent on the cortex: a process known as systems consolidation 16, 17, 18, 19. Whether all initially hippocampal-dependent memories are subject to consolidation is a point of some controversy 20, 21. Nevertheless, careful lesion studies in animals provide support for this hypothesis, suggesting that under some circumstances an offline process governs the modification of hippocampal memories, rendering them less susceptible to hippocampal damage 15, 22, 23, 24. Equally, a separate body of work points to a role for the hippocampus in planning and future thinking, that is, constructing potential scenarios. For example, patients with hippocampal damage have difficulty imagining the future [25] and are unable to describe rich fictitious scenes [26]. Moreover, functional magnetic resonance imaging (fMRI) indicates a distinct overlap between a network of brain areas, including the hippocampus, that are engaged during remembering as well as imagining events 27, 28.

**Figure 1:**
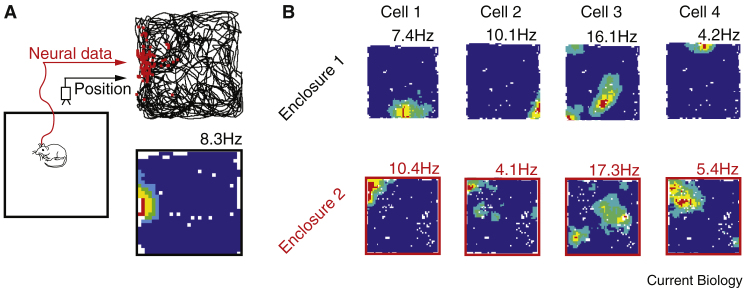
Place cells are characterised by their stable spatial firing fields. (A) Standard configuration for place cell recording. A rodent with chronically implanted extracellular electrodes forages in an open enclosure. Upper right: the animal’s path over the course of a 10-minute trial is indicated by the black line, action potentials from a single place cell are superimposed in red. Lower right: firing rate-map of the raw data indicating the mean firing rate of the cell per spatial bin. ‘Hotter’ colours indicate higher rates, peaking at 8.3 Hz (shown above the map); dark blue indicates low rates (0–20% of the peak rate); white bins are unvisited locations. (B) On exposure to an unfamiliar enclosure place cells ‘remap’, rapidly generating a novel representation; individual cells change their firing rate and field locations relative to each other and the environment 32, 64, 158. Recordings of four CA1 place cells (columns) made in similarly sized (70 cm square) enclosures located in different rooms (rows). Remapping is evident as a change in firing correlates and rates.

Electrophysiological investigations of the hippocampus and associated regions in rodents and other animals, including humans, have identified some of the key neural elements supporting memory and spatial cognition. Place cells, typically pyramidal neurons from areas CA1 and CA3 of the hippocampus, exhibit stable, spatially constrained firing fields, known as place fields 2, 29, 30, 31 (Figure 1). When an animal is in motion, the activity of a population of such place cells provides an accurate representation of its self-location 32, 33. Moreover, the fidelity of the place cell representation covaries with navigational accuracy, strongly implying that these cells are instrumental in guiding spatial decisions 34, 35. Subsequently, several complementary classes of neurons have been identified, representing other aspects of an animal’s position within the world: head direction cells, found throughout the limbic system, signal direction of facing 36, 37, 38; grid cells in the medial entorhinal cortex and para-subiculum represent self-location with an efficient periodic code 39, 40; and border cells as well as boundary vector cells signal proximity to elongated features of the environment 41, 42, 43. Clearly the representation of self-location provided by these neurons is likely to play a role in spatial memory [44]. It is also evident, however, that information about the animal’s current position alone is insufficient to account for either consolidation or the apparent role of the hippocampus in future thinking and navigational planning.

Interestingly, in the last twenty years, it has become clear that the activity of place cells do not simply represent an animal’s self-location but, under certain circumstances, also ‘replay’ past experiences 45, 46, 47 and potentially construct new spatial trajectories 48, 49, 50. Here we present a review of the replay literature and critically assess evidence supporting its hypothetical role in memory consolidation and planning. Further, we describe key questions for future studies to address, emphasising the need for novel behavioural tasks and the development of new technologies that can more precisely identify and perturb hippocampal activity patterns in order to provide firm evidence for a functional role of replay in mnemonic function and goal-directed behaviour.

### Reactivation of Past Place Cell Sequences

It has been known for some time that hippocampal neurons transiently increase their firing rate during sleep 51, 52. Indeed, in 1978, O’Keefe and Nadel [2] noted that, during quiescence and quiet wakefulness, the local field potential (LFP) of CA1 was interrupted by short bouts (40–120 ms) of high frequency (140–250 Hz) oscillations superimposed on lower frequency deflections; the high frequency components were termed ‘ripples’ and the composite signal is known as a sharp wave ripple 45, 53. It was during these sharp wave ripples that CA1 pyramidal cell activity was observed to dramatically increase [2]. A decade passed, however, before the link between this phenomenon and prior experience was identified, with the first demonstration that place cells which have been active during recent exploration are most likely to be reactivated during a subsequent sleep session [54].

Subsequent studies of hippocampal reactivations were aided greatly by the development of tools enabling concurrent recordings of large (>50) numbers of place cells. In this context, Wilson and McNaughton [45] recorded from 50–100 place cells per session while rats foraged on an elevated circular platform, as well as during prior and subsequent sleep sessions. They observed that place cell pairs which were temporally correlated during exploration also tended to exhibit correlated activity during the following period of sleep; in other words, cells with overlapping place fields fired together during rest. This relationship was not present in sleep recordings made before foraging. As such, they proposed that the preserved correlation between place cells represented a ‘reactivation’ of previous wakeful experience [45]. Importantly, correlated activity was most pronounced during sharp wave ripples. Subsequently, the same group [46] extended their work, showing that reactivations during sharp wave ripples explicitly recapitulated the relative timing of activity between cell pairs, strongly suggesting that sequences of spiking seen during awake behaviour were being reinstated; this effect was labelled ‘replay’.

**Figure 2:**
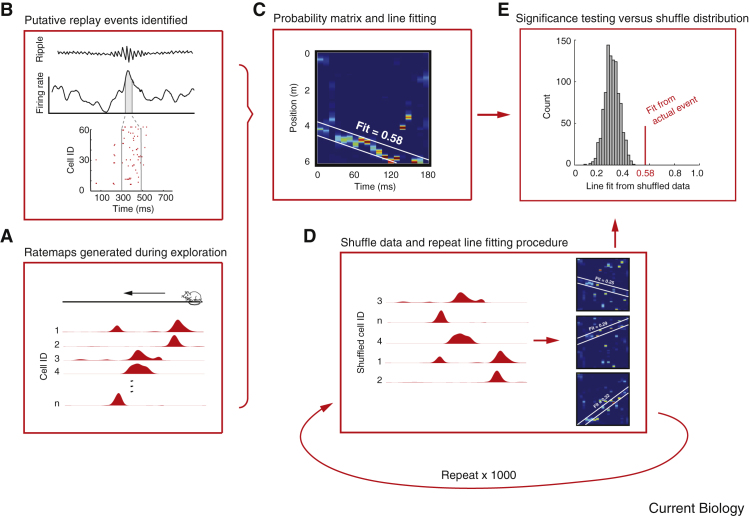
Typical methodology for detecting and analysing replay. (A) Linearised ratemaps are generated based on recordings made while rodents traverse a track. (B) In a subsequent rest period or during pauses in a task, hippocampal replay is marked by a high frequency ‘ripple’ oscillation in the LFP (top trace), which is associated with elevated multi-unit place cell activity, lasting ∼100 ms (middle, top). (C) A Bayesian approach is used to calculate, for each temporal bin (x-axis, typically 10 ms bins), the probability of the animal’s location on the track given the observed action potentials. Hot colours designate higher probability. A fitting procedure, typically enforcing a fixed velocity, is used to find the most likely trajectory encoded by the posterior probability matrix (shown in white, goodness-of-fit value indicated above the line). (D) To determine statistical significance a shuffling procedure is conducted. The cell ID of each place cell is randomly permuted such that the spike trains observed during the putative replay events are associated with different place fields. This process is repeated at least 100 times; on each iteration, the posterior probability matrix is recalculated and a goodness-of-fit for the best trajectory determined. (E) The goodness-of-fit obtained for the original event is ranked against the shuffled distribution, determining the probability of obtaining the goodness-of-fit value by chance.

Contemporary investigations of replay use similar methods, comparing the dynamic activity of cell pairs recorded during exploration with those observed during rest. Moreover, many recent studies explicitly compare sequences of place cells corresponding to entire trajectories, rather than the coactivation of cell pairs, effectively decoding the content of the replay event (Box 1 and Figure 2). To facilitate sequence matching, the experimental environment typically consists of a track on which animals can follow only relatively simple routes, reducing the awake experience to a limited number of place cell sequences that can be identified during replay 47, 55, 56. Lee and Wilson [47] were the first to apply this approach, recording place cells while rats ran back and forth on an elevated track. They showed that, during slow wave sleep, particularly in close temporal proximity to sharp wave ripples, replayed sequences of place cells corresponded to temporally compressed versions of trajectories that the animals had made in the preceding session. Indeed, the typical duration of a replay event varies between 100 and 300 ms 47, 56, 57, a ∼20 times increase in speed over the actual experience [58] — though replayed trajectories occurring during REM sleep progress at a more natural speed [59]. As with earlier work, replay of trajectories along the track were not detected in recordings made before the animal was placed into that environment.

Box 1 **Analysing replay.**

Replay analyses typically consist of three distinct phases: first, detection of putative replay events; second, decoding the spike activity recorded during the putative events; and third, identification of *bona fide* replay events representing trajectories through the animal’s environment.

The most common strategies for detecting putative events are either to identify the ripple component (140–250 Hz) of sharp wave ripples in the LFP 48, 64, 82, 156 or to directly detect brief periods (40–500 ms) when multi-unit firing rates are markedly elevated (more than three standard deviations above the mean, for example) 55, 56, 71, 95.

The content of putative replay events is then analysed by comparing the sequence of spikes emitted during the event with the sequence expected to arise as an animal traverses the environment (Figure 2, left). Because the vast majority of studies are conducted on tracks and runways, which constrain the animal’s movements, this is a tractable problem (though see [71]). Comparisons of the observed and expected spike sequences can be made using rank-order correlations (for example 50, 55, 56) but probability-based methods are more powerful. Commonly, the observed spike sequence is divided into short time windows (∼10 ms); Bayes formula with a uniform prior is then used to calculate the probability of the animal being at each position on the track given the observed spikes 33, 57, 64, 66, 95, 142, 157. Ratemaps, which define the expected firing rate of cells across the track, are generated during track running and firing rate probability distributions are assumed to be Poisson. Thus, each putative replay event results in a posterior probability matrix which expresses the probability of the animal’s location at different points in the environment as a function of time (Figure 2, centre).

Finally, replay events corresponding to trajectories through the recording environment are detected by assessing the extent to which the posterior probability matrix depicts incremental movement across some portion of the environment. Practically, this is often achieved by assuming that the replayed trajectory has a constant velocity, in which case a linear fit can be applied to the posterior probability matrix 57, 95; the goodness-of-fit then provides a measure of the extent to which a contiguous path is represented.

To determine significance and to control for differences in the number and distribution of spikes, this linear fit is compared against fits generated from shuffled data. A common strategy is to either randomise the identity of the cells from which the observed spikes originated or to randomise the location of the place fields on the track [57]. Data from each putative event are shuffled >100 times, each time calculating a posterior probability matrix, and establishing the best linear fit. The rank of the fit obtained for the experimental data relative to the shuffled population establishes the significance level (Figure 2, right). Different shuffling procedures effectively make different assumptions about what constitutes a valid null hypothesis and some shuffles are likely to be more conservative than others 95, 113. For example, permuting spike assignments such that spikes from the same cell are allocated to different cells assumes a degree of independence that is not present in biological data, and is likely to result in an overly liberal criterion for the identification of replay. One possible solution is to conduct several different types of shuffle, accepting only replay events that are identified reliably by all methods [57].

**Figure 3:**
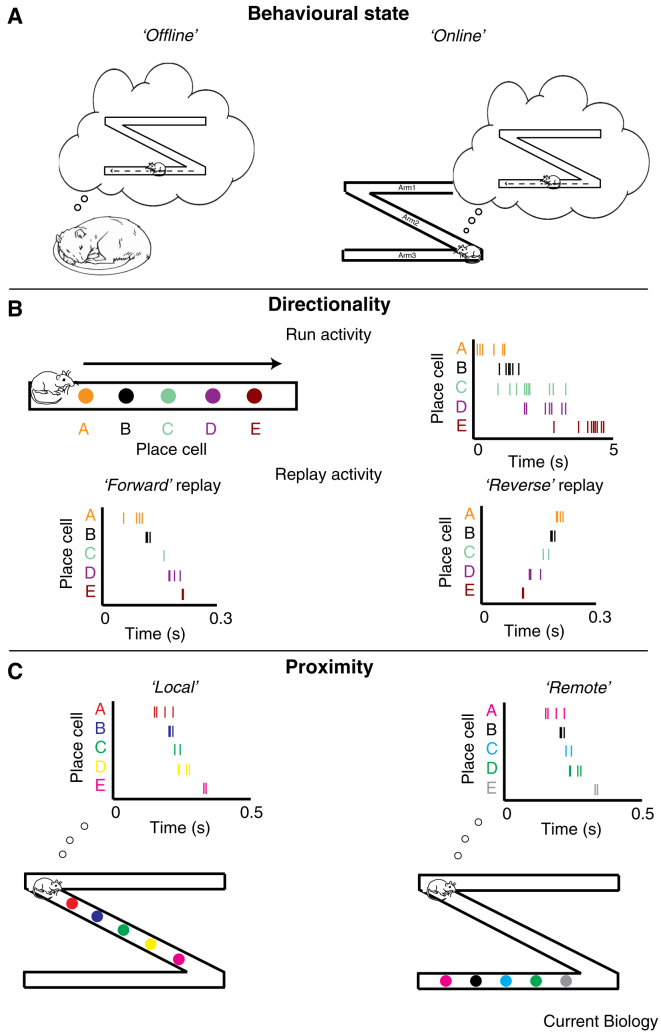
Replay types. (A) Replay occurs during rest/slow wave sleep (‘offline’ replay, left) 45, 47, 85 as well as during brief pauses in awake behaviours (‘online’ replay, right), such as when stopping at a decision point during navigation 48, 55, 56, 117. (B) Top: As an animal runs down a track, place cells are sequentially activated. Bottom: during replay, place cells can be reactivated in the same sequence as was experienced during running (‘forward’ replay, left) 46, 47 or in the opposite sequence (‘reverse’, replay, right) 55, 56, 66. (C) Online replay can depict locations proximal to the animal’s current location (‘local’ replay, left) 71, 85, 122 or be distant to the animal (‘remote’ replay, right) 63, 64, 122.

Subsequent work found that replay was not limited to ‘offline’ periods — during sleep or quiet rest — but could also be observed while animals were awake and engaging in simple tasks, such as eating, grooming, or while paused at decision points 55, 56, 60 (Figure 3A). For example, Foster and Wilson [55] found that replay trajectories were emitted when animals paused to consume a food reward at the ends of a track [55]. Although ‘online’, such replay shares many of the characteristics observed during offline periods: both occur while the animal is stationary and theta-band oscillations (6–12 Hz) are absent from the LFP (though see 61, 62 for examples of replay, or forward ‘sweeps’, during theta states while animals were located at decision points during a spatial task). Indeed, while sensory features of the current environment can shape the content of online replay — in larger environments, for example, the replayed trajectories tend to be longer [57] — that does not mean that it is a simple recapitulation of recent sensory experience. Like offline replay, it can encode paths remote to the animal’s current position 48, 57 (Figure 3C) or through enclosures entirely separate from the current one 63, 64. Finally, during both online and offline replay, the relative proximity of cells in the sequence is preserved 55, 56, 65, 66, but the absolute order can be reversed [55] (Figure 3B), inverting the sequence experienced during awake behaviour. Indeed, because place cells are typically directional on a linear track and therefore disambiguate travel in either direction 67, 68, the place cell sequences generated during ‘reverse’ replay are never observed during normal exploration.

Thus, a considerable canon of research describing the characteristic features of replay now exists. Although first identified during sleep, it is now evident that replay is not limited to offline states. The content of replay and conditions that modulate its occurrence seem to be equally diverse, and accordingly a number of distinct hypotheses describing a putative role for replay in mnemonic function and spatial cognition have been proposed. The two most prominent theories are that replay represents the mechanism underlying memory consolidation 45, 69 and that it may support planning during spatial decision making and goal-directed navigation 56, 70, 71, 72.

### Replay as Systems Consolidation

The role of the hippocampus in episodic memory indicates that it is particularly important for the rapid — ‘one-shot’ — learning of events and places [19]. However, theoretical studies indicate that this renders the hippocampal memory system prone to catastrophic interference, whereby new learning can rapidly disrupt or erase previously encoded memory traces [73]. Hence, a process of consolidation after the initial encoding of a new memory trace is essential to transform that trace from a temporary, labile state to a more stable and permanent form (see Squire *et al.* [74] for a recent review).

Interestingly, early neuropsychological data also indicated that the hippocampus plays a time-limited role in memory storage, best characterised by a temporal gradient of retrograde amnesia 1, 3. These findings led several authors 16, 17, 18, 19 to propose a theory of systems consolidation, whereby the ‘offline’ reactivation of hippocampal memory traces allows the gradual strengthening of complementary memory traces in the slower learning neocortex. Subsequent research, however, has both complicated the standard model of systems consolidation and indicated that memory consolidation processes extend far beyond simple hippocampus–cortex interactions.

First, evidence suggests that intracellular and intra-regional processes aimed at stabilising changes in synaptic strength also contribute to the consolidation of recently acquired or reactivated memory traces. In addition, there is some debate over whether memory traces ever become fully independent of the hippocampus, given that the hippocampus can be involved in the retrieval of both recent and remote memory traces 20, 75 and that memory consolidation appears to operate on a timescale of decades in humans 1, 3, 76. In either case, the selective strengthening of specific intra-hippocampal cell assemblies seems likely to play an equally important role in memory consolidation. Second, the rate of consolidation has been shown to depend on the amount of related prior knowledge that is available — the existence of appropriate schema 23, 77 — and it has been suggested that the process of consolidation acts to compress mnemonic information by extracting the principal statistical features from multiple unique experiences 24, 73. Hence, consolidation may be an active process by which new memory traces are selected and incorporated into the existing corpus of knowledge at variable rates and with differential success according to their content.

Whatever the specific nature and function of the memory consolidation processes may be, it seems clear that the reactivation of neural activity patterns at the population level following encoding is likely to contribute to the permanence of cell assemblies and therefore behaviourally relevant memory traces. Moreover, this reactivation is likely to occur during sleep or other quiescent states which provide relief from potentially interfering sensory activity, and involve neural activity on a timescale that is well-suited to the induction of synaptic plasticity and therefore the stabilisation of cell assemblies 78, 79, 80, 81. Given these requirements, Wilson and McNaughton [45] hypothesised that hippocampal replay might represent the neural mechanism supporting systems consolidation; and subsequent research has generated considerable, though not unanimous, support for this proposition.

If, as proposed, replay is important for stabilising behaviourally relevant memories, it might be expected that novel, recent, and salient experiences are preferentially reactivated: this appears to be the case. Replay, particularly reverse replay, more frequently represents novel as oppose to familiar environments [55]. This effect, measured by cell-pair co-activations, was found to be most pronounced on the first day of exposure to a novel environment and diminished on subsequent days [82]. Equally, during acquisition of a spatial alternation task, sharp wave ripples and related activity occurred more commonly after rats made a correct turn and were rewarded, as opposed to after unrewarded incorrect turns [83].

**Figure 4:**
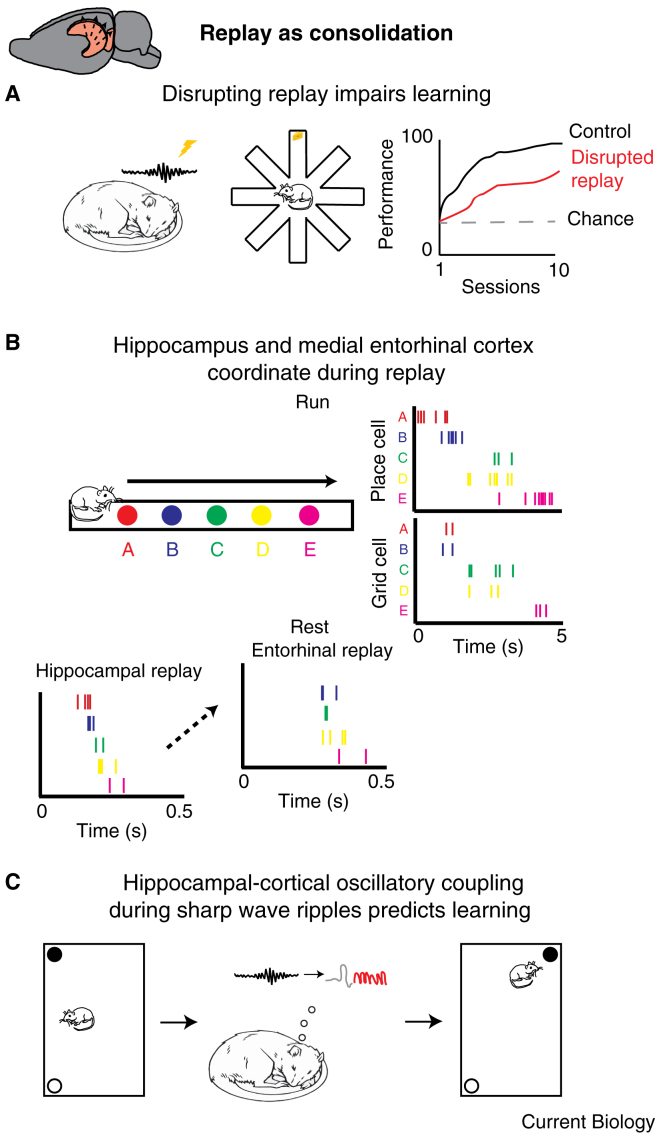
Replay as consolidation. (A) Sharp wave ripples in CA1 were detected and disrupted (via electrical stimulation) while animals slept (left) following training on a spatial reference memory task (learning the location of food on an eight-arm radial maze, middle). Animals acquired the task more slowly and consistently performed worse than control animals receiving stimulations outside sharp wave ripples (right) [69]. (B) Grid cells from the deeper layers of the medial entorhinal cortex and CA1 place cells were co-recorded while animals ran on linear runways (top) and during a subsequent rest session. Grid cell activity was significantly coordinated with place cell activity during hippocampal replay events recorded during rest, such that grid cells expressed similar spatial positions to place cells during replay (bottom) [95]. (C) Rats encoded the location of two objects in a rectangular arena (left), sharp wave ripples (from CA1) and delta-spindle sequences (from medial prefrontal cortex) were recorded during subsequent sleep (middle). The co-occurrence of hippocampal and cortical rhythms was associated with memory of the object locations, indexed by preferential exploration of a displaced object in the post sleep session (right). If the duration of encoding was shortened to impair learning, consolidation could be rescued by stimulating the cortex, when sharp wave ripples were detected, thereby inducing delta-spindle sequences [103].

In a similar vein, Csicsvari and colleagues 84, 85 demonstrated an explicit link between sharp wave ripple associated hippocampal reactivations and animals’ subsequent performance on a spatial memory task. Specifically, rats were required to remember the location of multiple unmarked food wells and navigate quickly between them. The number of goal location reactivations during both rest and task engagement predicted performance; though a causal relationship was not established. Ensuing studies went further, however, and showed that the electrical interruption of sharp wave ripples, and presumably replay, reduced the rate at which animals acquired a spatial memory task 69, 86 (Figure 4A). Hence there is a compelling, albeit incomplete link between the occurrence of replay and successful retention of information. Nonetheless, the location and extent of plasticity being instantiated by replay remain unclear.

The standard theoretical view of systems consolidation has focused on the diminishing requirement for hippocampal involvement in memory retrieval that occurs with time [16] whereas alternative explanations assume a sustained role for the hippocampus in conjunction with intra-hippocampal reorganisation of memories [21]. While it is too early to settle this long-standing debate, it is clear that replay is well placed to generate offline changes in both hippocampal and cortical memory networks. In the Csicsvari group studies 84, 85 described above goal location reactivations were observed to co-occur with a clustering of CA1 place fields around the goal, though curiously not those in CA3, suggesting that offline replay shapes hippocampal memories. This link was made explicit by recent work [87] in which the activity of a sub-population of CA1 place cells was disrupted during sharp wave ripples and the stability of the manipulated place fields alone was reduced, although in this case the manipulation was made while animals remained in the test environment.

It is also clear that sharp wave ripple associated activity propagates from the hippocampus to neighbouring structures such as the pre-subiculum and para-subiculum, as well as medial entorhinal cortex 88, 89. In fact, a number of authors 90, 91, 92 have investigated similarities between activity patterns during wakefulness and quiescence in various extra-hippocampal regions. These studies have frequently used an ‘explained variance’ [92] approach to identify reactivations, that is, a measure of the degree to which activity patterns observed during post task rest can be accounted for by wakeful activity patterns. Recording from the ventral striatum while rats performed T-maze spatial alternation, Pennartz *et al.* [92] found significant reactivation of wakeful activity patterns during post task sleep. Many ventral striatal cells were also modulated by hippocampal sharp wave ripples, and those that were showed stronger reactivations. Similarly, recordings made in macaques during and after a sequential reaching task found significant reactivations in posterior parietal, motor, somatosensory and dorsal prefrontal cortex [90]. The authors also found evidence for reactivations of wakeful inter-regional activity patterns.

A smaller number of studies 93, 94, 95, 96 have explicitly shown that coordinated cortical-hippocampal activity associated with awake behaviours is present during replay events. Ji and Wilson [93] were the first to demonstrate this using simultaneous recordings from visual cortex (V1) and hippocampus made while rats ran on a track. They observed many V1 cells that had spatial firing fields on the track, resulting in distinct sequences of activity which were replayed during a subsequent sleep session. Moreover, replay of similar trajectories in hippocampus and V1 tended to co-occur [93]. To be consistent with the simple consolidation hypothesis, however, one would expect hippocampal replay to invariably precede cortical replay; in fact, in this study it was not possible to establish if replay was initiated in the hippocampus and, although replay in the two regions did coincide, in some cases it appeared that V1 trajectories were generated with no hippocampal contribution. This result may simply reflect the difficulty of making population level inferences from a relatively small number of recorded single units. Alternatively, it might point to some form of bidirectional control over replay or suggest that, even during rest, the role of replay extends beyond simple consolidation.

Subsequently, other authors have applied similar methods to identify coordination between the hippocampus and prefrontal cortex [94], auditory cortex [96], and grid cells in the deep layers (V and VI) of the medial entorhinal cortex [95] (Figure 4B) — the principal cortical projection target of the hippocampus 97, 98. In the case of the study by Olafsdottir *et al.* [95], during rest, replay in the medial entorhinal cortex was seen to lag CA1 by around 10 ms; consistent with a hippocampal initiation of replay. Interestingly, a similar study examining activity during sharp wave ripples in superficial layers of the medial entorhinal cortex found quite different results: entorhinal replay frequently occurred without coordinated activity in the hippocampus [99]. Further, recent work from Yamamoto and Tonegawa [100] found that input from layer III of the medial entorhinal cortex was required for extended awake CA1 replay. In part, these findings may be accounted for by anatomical differences as superficial entorhinal layers are primarily an input to the hippocampus 97, 98. Equally, the latter two analyses were predominantly based on recordings made while animals were engaged in spatial tasks, and the observed replay may therefore be more strongly related to spatial planning and less to consolidation processes, in turn suggesting a differential involvement of superficial and deep medial entorhinal cortex layers in planning and consolidation, respectively.

Clearly then, there is ample evidence for coordinated cortical-hippocampal activity during sharp wave ripples, albeit with more complex dynamics than might be expected from the simplest model of systems consolidation. Still, if hippocampal replay activity is involved in systems consolidation processes, then one might expect replay to be associated not only with increased activity in cortex, but also with increased plasticity. Several authors (for example [81]) have noted that the rapid sequences of place cell activity observed during replay are optimal for inducing plasticity in post-synaptic targets. Although direct evidence for this is currently lacking, cortical oscillations that strongly modulate the depolarisation of principal neurons have also been associated with hippocampal sharp wave ripples during sleep.

Two of the main oscillatory events are delta waves [101], slow 0.1–4 Hz oscillations observed in a variety of cortical regions; and thalamo-cortical spindles 102, 103, 10–20 Hz oscillations originating in the thalamus. These two oscillatory patterns are often observed in close temporal proximity to each other and, importantly, to sharp wave ripples in the hippocampus 104, 105, 106, 107. Indeed, Maingret and colleagues [103] found that the successful recall of object locations following sleep was associated with enhanced coupling between hippocampal sharp wave ripples, cortical delta-waves, and spindle events (Figure 4C). Furthermore, when the duration of encoding was shortened to impair learning, the authors saw that performance could be rescued by stimulating the prefrontal cortex when hippocampal sharp wave ripples were detected, thereby generating delta waves and spindles. Similarly, it is clear that plasticity related to the spatial content of replayed trajectories can be induced during rest. This was beautifully demonstrated by de Lavilleon *et al.* [108]: During sleep, though not just during periods of sharp wave ripples, reward in the form of medial forebrain bundle stimulation was triggered whenever a specific CA1 place cell was active; subsequently, during exploration, animals spent more time in the place field of the corresponding cell.

While the foregoing discussion makes a convincing case for a role of hippocampal replay in memory consolidation, several important caveats must be considered. First, the majority of studies examining the modulation of replay by novelty, recency, and saliency have done so while animals are awake and engaging in tasks (for example 55, 82, 83). In contrast, the reduced sensory interference present during offline states are believed to render them favourable for consolidation. Nevertheless, this does not preclude the possibility that replay occurring during brief pauses in active behaviour also contributes to consolidation.

Second, the disruption of sharp wave ripples has been shown to impair spatial learning 69, 86, but it remains to be seen whether this effect is due to the disruption of replay specifically or simply due to disruption of the sharp wave ripple state. For example, stimulation during sharp wave ripples — when neurons in CA1 are highly active 2, 81 — may simply disrupt existing spatial memory traces in the hippocampus (for example [87]) rather than interfere with consolidation.

Third, although replay may be important for learning, whether it genuinely represents the mechanism supporting systems consolidation, or simply reflects an intra-hippocampal process stabilising newly formed memory traces, remains to be established. A convincing demonstration of the former would be to identify the cortical correlates of a hippocampal memory and show how these develop or mature with the occurrence of replay. This might be achieved, for example, using a similar method to Kitamura *et al.* [109] in which *c-Fos* expression was used to identify a cortical engram, hence determining its involvement in memory retrieval at different time points following learning. Alternatively, confirmation that memories do not become hippocampus-independent if replay is prevented from propagating to cortex would be compelling evidence. To address this latter point, it seems plausible that the interruption of sharp wave ripples could be used in conjunction with an approach similar to that used by Tanaka *et al.* [110]. Namely, an experimenter would probe the point at which reinstatement of a cortical representation occurred independently of the hippocampus, that is, after CA1 silencing.

Fourth, recent studies by several groups 50, 111, 112 have shown that replay can be observed even before animals have any experience of an environment. In other words, place cell sequences recorded during rest are subsequently found to correspond to paths through a novel environment. This ‘preplay’ is a controversial topic, and there is some disagreement regarding its existence [113]. Potentially, the effect might arise due to a failure in the statistical assumptions used to determine the significance of the observed sequences [113] (Figure 2). For example, if the place cell representation of the novel environment is not orthogonal to familiar environments, then replay of those familiar places might be construed as preplay. Equally, though, preplay may result from activity in preconfigured cell assemblies, possibly supported by attractor networks, that are primed for inclusion into future spatial representations 111, 112, 114, 115. Either way, preplay seems to be a weaker phenomenon than replay [112]; indeed, the level of correlation in place cell activity that existed prior to exploration of a novel environment was used as a control condition in early replay studies 45, 47. Hence, replay can still be understood as a process that predominantly reflects prior experience, though the extent to which it is influenced by the pre-existing configuration of hippocampal networks remains to be seen.

### Replay as Planning

The fact that replay occurs during awake behaviour suggests that, beyond its potential role in learning, it might also provide a mechanism for guiding navigational decisions, planning goal-directed trajectories or simulating the outcome of a given choice (for example 56, 70, 71, 72, 116, 117). This view was established by Diba and Buzsaki’s [56] observation that, during track running, replay occurring as rats stopped for reward at the track’s ends tended to be in the reverse direction, while movement initiation was mainly preceded by forward replay. The authors posited that reverse replay was important for learning from recent experiences, while forward replay was required for planning forthcoming actions. Although the functional distinction between forward and reverse replay is no longer clear cut (for example [65]), broadly similar results have been noted elsewhere. For example, as animals pause at a junction on a working memory task, replay events tend to depict routes ahead of the animal [117]. Similar forward ‘sweeps’ have been observed by others (for example 61, 62, 118), yet in these instances the sweeps seem to occur during theta states rather than sharp wave ripples. Although theta sweeps have been proposed to support decision making about immediate actions [61], it remains to be seen whether and how they differ functionally from forward replay observed during awake behaviour. Finally, various theoretical propositions suggest replay as a candidate mechanism for exploring potential routes or extracting goal-directed heading vectors 72, 116, 119.

If replay does represent a mechanism for planning spatial trajectories, then its occurrence should, in general, predict accurate behaviour. Indeed, one might specifically expect replayed trajectories to prefigure the path an animal is about to take. To a certain extent this appears to be true. For example, an increase in the probability of cell-pair co-activation during sharp wave ripples has been seen to precede correct turns on a W-maze and, in the same experiment, a non-significant trend was noted for preferential replay of the ‘correct’ arm [117]. Similarly, when animals were engaged in open field navigation to a hidden goal, Pfeiffer and Foster [71] found a tendency for replay trajectories to propagate in the direction animals were about to travel — an effect that was not present when the rats were foraging randomly. The analysis did not, however, establish whether the replayed trajectory predicted the exact path the animal was about to take. Indeed, in the absence of a clear mechanism linking replay and navigational guidance it is not obvious if such a relationship really should be expected.

Several groups 72, 116, 119 have proposed, based on theoretical analyses, that replay in entorhinal grid cells provides the means to calculate goal-directed vectors. Current models, though, are agnostic as to how place cells should respond during bouts of grid cell replay; indeed, recent experimental work suggests that grid cells and other spatial cell types in the superficial layers of the medial entorhinal cortex exhibit replay independently of CA1 [99]. More broadly, if online replay does provide a mechanism for planning trajectories, then one might occasionally expect to observe novel sequences of activity; for example, corresponding to a new combination of turns through an environment. This does appear to occur: in fact, replayed sequences have been reported to combine elements of an environment that were experienced separately and even to represent paths through a section of the environment the animal had seen but not experienced 48, 49. Indeed, in the latter case, despite the fact that replay was recorded during rest, activity was seen to preferentially represent a section of the environment leading to reward, implying a link with planning [49].

**Figure 5:**
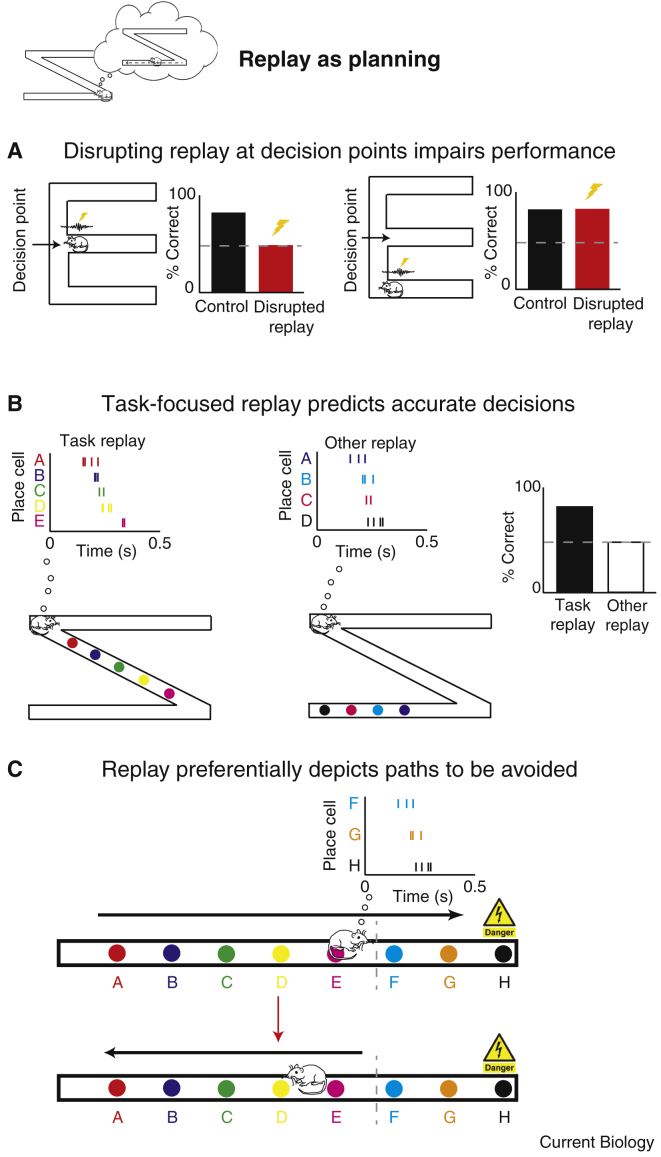
Replay as planning. (A) Disrupting sharp wave ripples at decision points in a spatial alternation task (‘W maze’) was associated with impaired performance compared to control animals (left). When sharp wave ripples were disrupted at non-decision points performance was preserved (right) [120]. (B) Replay was recorded at the corners of a Z-shaped track preceding correct and incorrect turns. When replay depicted positions consistent with the animals’ current positions (for example, proximal locations, left) the rats were more likely to make the correct turn (right). Whereas if replay depicted positions not immediately relevant to current behaviour (middle), animals were less likely to make the correct turn (right) [122]. (C) Following training on an inhibitory avoidance task (learning to associate the end of a linear runway with a foot shock), replay during pauses preceding entry to a shock zone preferentially depicted paths towards the feared zone (top) and was associated with the animals turning away from the shock zone and running in the opposite direction (bottom) [121].

More generally, normal hippocampal sharp wave ripple-related activity is necessary to support spatial decision making under some circumstances. Clear evidence for this was provided by Jadhav and colleagues [69] using closed-loop electrical stimulation of the ventral hippocampal commissure to truncate sharp wave ripples while animals performed a W-maze alternation task. Interruption of the sharp wave ripples decreased performance and the rate at which the task was acquired [120] (Figure 5A). Despite causally connecting sharp wave ripple-related activity and spatial decision-making, the specific mechanism linking replay with behavioural output is still unclear, and so it is hard to discern the functional contribution of replay in this scenario.

As intimated above, one of the central difficulties in ascribing a specific function to ‘online’ replay has been the fact that the trajectories depicted do not obviously relate to ongoing behaviour, often representing non-local positions 48, 57, 63, 64 de-coupled from the animal’s future path [121]. For example, when animals were required to run laps on one section of a track, Gupta *et al.* [48] observed that replay trajectories preferentially represented portions of the maze that were not currently in use and which had not been recently visited. Similarly, recent work [121] has demonstrated preferential replay of paths that animals were incentivised to avoid, and these trajectories were most often replayed prior to animals turning away from the aversive region and hence were anti-correlated with subsequent behaviour (Figure 5C).

In part, some of these apparent discrepancies can be accounted for by immediate behavioural relevance to the animal, with replay providing a mechanism by which prior associations or memories are recalled and used to direct behaviour. But online replay is also known to depict trajectories through environments distinct from that in which the animal is currently located, which were experienced earlier in the same recording session [64] or even on previous days [63]. It is more difficult to envisage the immediate behavioural relevance of a distant environment or indeed even for replay in the absence of an ongoing task. What could account for these divergent findings?

A viable hypothesis seems to be that, in part, this variability arises because ongoing task demands influence the nature and content of replay. Namely, during periods characterised by a low cognitive load when animals are not engaged in a demanding behavioural task — such as sitting still or shuttling back and forth on a linear track — replay occurs in order to support the consolidation of existing memories, as it does during slow wave sleep. Conversely, when animals actively engage in a task, such as at a decision point, replay becomes task focused. Consistent with this view, O’Neill *et al.* [84] observed that shortly after animals arrived at a reward site replay was task-focused, preferentially expressing the goal location; whereas, when animals lingered at the site — disengaging from the task — replay was more likely to express places remote from the current position.

Similarly, recent work from our own laboratory [122] examined the dynamics governing this switch. We saw task-related replay immediately as animals arrived or departed from a decision point, depicting forward trajectories focused on the current location. Conversely, when animals remained at the decision point, replay changed to preferentially encode trajectories in both forward and reverse directions that were distributed across the apparatus, rather than concentrated on the current location; suggestive of consolidation processes. In addition, we found that accurate spatial choices tended to be preceded by forward replay focused on the animal’s current position, as one would expect if these dynamics contributed to spatial decision making (Figure 5B).

Furthermore, even when animals are actively engaged in a task, the observed replay is expected to be variable. Although replay under these circumstances might be task related, this does not mean that replay should exclusively depict, and consequently predict, future paths. If adaptive behaviour requires the retrieval of past actions or experiences then replay might equally, or indeed preferentially, express prior behaviour. This proposition could account for the varied findings relating to online replay (for example 48, 71, 84, 117, 121). In this context, replay may be considered to mediate the retrieval of information needed for accurate behaviour, rather than planning of a specific trajectory; and any distinction between online and offline replay is not particularly useful. ‘Offline replay’, typically recorded while an animal is either sleeping or resting in a holding environment, is likely to consist almost entirely of consolidative replay. ‘Online replay’, on the other hand, will be mixed; varying between task-relevant and consolidative replay according to the animal’s current behavioural state and motivation.

To summarise, the content of online replay has been found to be surprisingly variable. Although it may support spatial planning under some conditions, equally often it seems to serve other functions. We suggest that an animal’s engagement with ongoing task demands may account for some of these differences. When engaged in a task, replay contributes to spatial decision making and navigation, and hence is largely focused on the animal’s current milieu. In the absence of specific task demands, replay subserves consolidation, meaning the content of replay will likely be more varied, potentially representing the full range of the animal’s recent experiences.

### Replay in Human and Non-human Primates

Because of the relative paucity of single unit electrophysiology studies outside of the rodent, there is limited evidence for the existence of hippocampal replay in humans and non-human primates, though a number of studies have reported spatial and non-spatial firing correlates of individual neurons in hippocampal and parahippocampal regions that would permit such analyses in future 30, 123. Nonetheless, several intracranial recording studies have characterised sharp wave ripple activity in both the human 124, 125, 126 and primate 127, 128 hippocampal LFP. These ripples appear at a frequency of approximately 100 Hz (lower than that typically observed in the rodent), have a duration of around 50 ms, are observed during both quiet rest and non-rapid eye movement (NREM) sleep and tend to co-occur with, and be phase locked to, cortical slow waves 126, 128, 129, 130. Only a few studies have co-recorded single unit activity, however, showing that burst firing activity is increased in the human hippocampus during slow wave sleep [131], and in primate CA1 during sharp wave ripples [127], but not examining the finer temporal structure of that activity.

Alternative approaches have provided inferential evidence for the replay of previous experience in the human brain during quiet rest and sleep. The best example comes from applying multivariate pattern classification techniques to magnetoencephalography (MEG) recordings, allowing the offline reoccurrence of neural activity patterns associated with different visual stimuli to be identified [132]. Using this technique, it has been demonstrated that sequences of stimuli initially encoded during a non-spatial navigation task were reactivated in reverse order on a timescale consistent with hippocampal replay during a subsequent 30 second planning period, although the signal appeared to originate from occipital/posterior temporal sources.

Similar methods, applied to fMRI data, have shown that, during rest, the frequency with which stimulus representations are reactivated correlates with subsequent memory performance 130, 133. Furthermore, other neuroimaging studies have shown that manipulations which bias replay in rodent models — i.e. the presentation of sound or odour cues during sleep — generate an increase in the hippocampal BOLD signal 134, 135, 136. Moreover, these interventions can enhance subsequent memory performance (but see van Dongen *et al.* [137]), and the memory benefit correlates with hippocampal volume [138].

Interestingly, one study [126] showed that the number of sharp wave ripples in the human medial temporal lobe during a short rest period after learning correlated with mnemonic performance. Importantly, however, none of these studies provide direct evidence for hippocampal replay. Moreover, similar performance benefits are seen on procedural tasks that are not hippocampal dependent, suggesting that these interventions may access a more general mechanism of memory enhancement [139]. Hopefully, future studies, making use of single-unit data derived from patients with intra-cranial depth electrodes, will be able to investigate the existence of replay in humans; providing an important translational connection between rodent and human hippocampal research.

### Future Directions

Replay is known to occur during sleep, rest and active navigation, and has been ascribed a number of functional roles including the stabilisation of newly formed memories, planning and decision-making during spatial tasks. In general, there is strong support for each of these propositions, although the relationship is more complex than was originally envisaged and specific mechanisms are still lacking. Indeed, a number of clear caveats exist.

First, to assess whether replay supports systems consolidation, it is not sufficient to simply manipulate hippocampal replay (or indeed sharp wave ripples) and assess the effects on behaviour. Rather, it is also necessary to demonstrate that this manipulation either enhances or delays the maturation of memory traces, be they in the hippocampus or cortex.

Second, the relationship between awake replay and ongoing behaviour is complex. In part this likely reflects the fact that, during awake periods, replay might support consolidation as well as task-related processes. Further, even when replay appears to be related to an ongoing task, the specific link between replayed trajectories and subsequent behaviour is not trivial. A fuller understanding of the network mechanisms that control the apparent ‘switch’ between replay for consolidation and replay for planning will resolve some of this variability. Beyond this, untangling the relationship between replay and behaviour will also require carefully constructed behavioural tasks with clearly delineated demands in which performance depends on defined and spatially localised sources of information (see for example [66]).

Third, the role played by extra-hippocampal regions in the generation and control of replay has received relatively little attention. Both the medial septum and mesopontine median raphe region have been shown to modulate the occurrence of sharp wave ripples 140, 141, but the extent to which these regions govern the occurrence of replay during the course of normal behaviour is unknown. Similarly, it appears that cortical regions have a greater autonomy to initiate and potentially guide replay than was previously imagined (for example [99]), but again, the behavioural relevance of cortical replay and how it relates to hippocampal replay requires further study. More specifically, the mechanism controlling which precise sequence of place cells is replayed is also unknown.

One possibility described above is that cortical activity, possibly itself resembling replay, might prompt the reactivation of hippocampal sequences (for example 93, 99). Consistent with this view, it is known that presentation of familiar auditory stimuli during sleep biases hippocampal replay to represent the locations in which those stimuli were encountered during wakefulness [142]; presumably auditory cue presentation causes the reinstatement of stimuli-specific activity patterns in the auditory cortex, ultimately promoting activity in place cells associated with that cue during training.

An alternative mechanism is provided by dopamine signalling from the ventral tegmental area (VTA) [143] and locus coeruleus [144]. Dopamine signalling in the hippocampus is known to be important for the stabilisation of place cell activity [145] and the retention, but not initial encoding, of spatial memories [146]. Indeed, optogenetically triggering dopamine release in mice during exposure to a novel environment results in stronger CA1 reactivations of that environment during rest and a concomitant increase in memory retention [147]. However, it is not known whether dopamine directly modulates the occurrence of replay or whether the apparent increase in reactivation results from other mechanisms that stabilise CA1 activity. Interestingly, VTA neurons were found to have elevated firing rates during sharp wave ripples emitted while animals conducted a spatial task, but not during sleep [148]. This is particularly relevant because VTA neurons are known to represent reward prediction error [149] and are central for reinforcement learning [150]. In turn, reverse replay during behaviour has been implicated as a possible solution to the temporal credit assignment problem in reinforcement learning 55, 70. The juxtaposition of VTA activity and replay supports this general view but, again, does not necessarily implicate dopamine as a modulator of replay itself.

Lastly, evidence for the existence of replay outside of the rodent is at best indirect. It will be exciting to see if emerging technologies in human intracranial recordings are able to resolve large populations of single neurons — and thus, replay — in the human brain. Such studies would provide essential insight into potential similarities between rodent and human hippocampal function and clarify the hypothesised role of hippocampal replay in mnemonic functions and goal-directed behaviour.

In general, looking to the future, it appears that several technical developments are of particular relevance to this field. Historically one of the problems in studying the long-term effects of replay on hippocampal and cortical networks has been the difficulty in stably recording large populations of neurons for significant periods of time. The increasing availability of two-photon calcium imaging coupled with rodent virtual reality now means that activity of hundreds of cells can be monitored across days, while animals perform spatial tasks [151]. Indeed, the temporal resolution of the most recent generations of calcium indicators is sufficient to allow replayed sequences to be detected optically [152], and so it is already possible to monitor the incremental changes induced in a hippocampal network as a result of replay. Going further, the combination of optogenetics and optical imaging, allowing for the rapid manipulation of visually identified cells, provides a powerful means of perturbing, or even artificially generating, specific replay sequences [153]. Such an approach would provide the means to explicitly link replay of a sequence with specific mnemonic or behavioural outcomes. To be viable though, this would likely require a control system triggered by the rapid decoding of replay trajectories, and some progress towards this goal has already been made 154, 155.

### Conclusions

To conclude, research over the past twenty-five years has contributed to understanding the role hippocampal replay plays in cognition. Current evidence suggests that replay is used to selectively strengthen newly acquired memories for retention and guide adaptive decisions during active behaviour. Yet, numerous questions regarding the underlying mechanisms remain. Does replay mediate the maturation of cortical memories, stabilise newly formed hippocampal cell assemblies, or instigate changes in both regions? Is the content of replay prescribed or can it adapt to changing demands, dictated by current tasks and behavioural state? Newly available techniques are well placed to address these questions, and it seems likely that the next decade will reveal a clearer understanding of the functional roles replay performs, the mechanisms by which it acts, and the systems by which it is controlled.
